# CD160Ig Fusion Protein Targets a Novel Costimulatory Pathway and Prolongs Allograft Survival

**DOI:** 10.1371/journal.pone.0060391

**Published:** 2013-04-04

**Authors:** Francesca D’Addio, Takuya Ueno, Michael Clarkson, Baogong Zhu, Andrea Vergani, Gordon J. Freeman, Mohamed H. Sayegh, Mohammed Javeed I. Ansari, Paolo Fiorina, Antje Habicht

**Affiliations:** 1 Transplantation Research Center, Renal Division, Brigham and Women’s Hospital and Children’s Hospital Boston, Harvard Medical School, Boston, Massachusetts, United States of America; 2 Transplantation and Internal Medicine Division, San Raffaele Scientific Institute, Milan, Italy; 3 Department of Renal Medicine, Cork University Hospital, Cork, Ireland; 4 Department of Medical Oncology, Dana Farber Cancer Institute, Boston, Massachusetts, United States of America; 5 Transplant Center Munich - LMU, University Hospital, Munich, Germany; University of Tor Vergata, Italy

## Abstract

CD160 is a cell surface molecule expressed by most NK cells and approximately 50% of CD8^+^ cytotoxic T lymphocytes. Engagement of CD160 by MHC class-I directly triggers a costimulatory signal to TCR-induced proliferation, cytokine production and cytotoxic effector functions. The role of CD160 in alloimmunity is unknown. Using a newly generated CD160 fusion protein (CD160Ig) we examined the role of the novel costimulatory molecule CD160 in mediating CD4^+^ or CD8^+^ T cell driven allograft rejection. CD160Ig inhibits alloreactive CD8^+^ T cell proliferation and IFN-γ production *in vitro*, in particular in the absence of CD28 costimulation. Consequently CD160Ig prolongs fully mismatched cardiac allograft survival in CD4^−/−^, CD28^−/−^ knockout and CTLA4Ig treated WT recipients, but not in WT or CD8^−/−^ knockout recipients. The prolonged cardiac allograft survival is associated with reduced alloreactive CD8^+^ T cell proliferation, effector/memory responses and alloreactive IFN-γ production. Thus, CD160 signaling is particularly important in CD28-independent effector/memory CD8^+^ alloreactive T cell activation *in vivo* and therefore may serve as a novel target for prevention of allograft rejection.

## Introduction

Full T cell activation in response to alloantigen presentation requires, in addition to stimulation through the TCR, a costimulatory signal [1]. One of the most widely studied positive costimulatory signals is the CD28:B7 pathway. While CD28 blockade has been shown to prolong allograft survival in some transplant models [2]–[6], less success has been observed in more stringent models [7]. This process is thought to be mediated predominantly by NK cells [8]–[9], CD8^+^ T cells [10]–[13] and effector/memory responses [14], which appear to be less dependent on CD28 costimulation, and might therefore utilize alternative costimulatory pathways for activation [15].

CD160, is an immunoglobulin (Ig)-like glycosyl-phosphatidylinositol (GPI)-anchored cell membrane receptor, which was first identified in humans [16]–[18]. The murine expression of CD160 is similar to that in humans and is largely restricted to cells with cytolytic activity including NK cells, NKT cells and activated CD8^+^ T cells [17], [19]–[20]. Furthermore, CD160 is expressed by growing endothelial cells and blocking its interaction with endothelial cells using a human anti-CD160 mAb (CL1-R2) has been shown to diminish angiogenesis without the need for Fc receptor–bearing cytotoxic immune cells [21].

Both classical and non-classical MHC class-I molecules, including CD1d, bind to CD160 with low affinity [16], [19], [22]–[23]. Recently HVEM was identified as the signaling ligand for CD160 [24]. CD160 binds HVEM in the CRD1 region [25] forming a disulfide-linked interchain homotrimers [26] that can activate HVEM signaling, and form a bidirectional signaling pathway [25]. Crosslinking murine CD160 enhances NK and T cell cytolytic activity [16], [22]–[23] and leads to the production of IFN-γ, TNF-α and IL-6 [22], [27]. CD160 particularly stimulates CD8^bright^ cytotoxic effector lymphocytes lacking CD28 expression [16], CD8^+^CD160^+^ memory T cells [20] and seems to deliver a potent costimulatory signal to activated but not naïve CD8 T cells [28]. However, engagement of CD160 on human CD4+ T cells by HVEM has been shown to block LIGHT-HVEM induced activation [24] and cross-linking of human CD160 with mAb inhibit anti-CD3/anti-CD28-induced CD4 and CD8 T cell activation profoundly [29]. However, the CD160-mediated inhibition of human T cell proliferation has not been reproduced in murine T cells using rat anti-mouse CD160 mAb [16], [19], [22]–[23]. Furthermore, soluble CD160 blocks NK cell cytolytic activity [26], while the CD160 transmembrane form has an activating role in NK cells [30]. Thus, it seems that depending on the extracellular domain of the CD160 protein that is involved in the interaction, a costimulatory or coinhibitory signal can be transduced.

To date the role for the CD160:CD160L interaction in alloimmune responses is undefined. Since ligation of CD160 triggers NK and CD8^+^ T cell activation and effector function, we speculated that it might function as an alternative costimulatory signal particularly in CD28-independent allograft rejection. Using a novel fusion protein CD160Ig we demonstrate that blocking the CD160:CD160L interaction prolongs fully mismatched heart transplant survival in a model of CD28-independent CD8^+^ mediated allograft rejection by inhibiting alloreactive CD8^+^ T cell proliferation, effector/memory expansion and cytokine generation.

## Materials and Methods

### Mice

BALB/c (H-2^d^), B6.C-*H2^bm1^*/ByJ (H-2^bm1^) and C57BL/6 wild type (WT), CD28^−/−^, CD8^−/−^ and CD4^−/−^ knockout (KO) mice (all H-2^b^) were purchased from The Jackson Laboratory. All mice were used at 6–12 weeks of age.

### Ethics Statement

Mice were housed in accordance with Institutional Animal Care and Use Committee (IACUC) approval. All the experimental procedures were performed in accordance with institutional and National Institutes of Health guidelines and Institutional Animal Care and Use Committee (IACUC) approval. The study was carried out in strict accordance with the recommendations in the Guide for the Care and Use of Laboratory Animals of the National Institutes of Health. All surgery was performed using CO2 overdose euthanasia followed by thoracotomy, consistent with the recommendations of the American Veterinary Medical Association in accordance with Institutional Animal Care and Use Committee (IACUC) approval and all efforts were made to minimize suffering.

### Murine Heart and Skin Transplantation

Heterotopic vascularized cardiac grafts from BALB/c donors were transplanted using microsurgical techniques as described by Corry et al. [31]. Graft function was monitored by daily palpation. Rejection was defined as complete cessation of cardiac contractility.

Full-thickness trunk skin grafts from bm1 donors (∼1 cm^2^) were transplanted onto the dorsal thorax of recipient mice, sutured with 4-0 silk, secured with dry gauze and a bandage for 7 days. Skin graft survival was monitored daily. Rejection was defined as complete graft necrosis.

### CD160 Fusion Protein

The murine CD160Ig fusion protein was made by linking the complete extracellular domain of murine CD160 to the hinge-CH2-CH3 domain of murine immunoglobulin G 2a (mIgG2a) with four point mutations blocking Fc receptor and complement binding. Briefly, the complete extracellular domain of murine CD160 was amplified by PCR using a murine CD160 cDNA (C57/BL6 AK042093) as template and forward primer 5′- GCTACGGGTACCACCATGCAAAGAATCCTGATGGCCCCTG and reverse primer, 5′-GGCCCTTCCGGAGAGAGTGCCGTTGATATGGCTGAAG. The PCR product was digested with Acc65I and BspEI and cloned into Acc65I, BspEI digested mIgG2a-pEF6 vector. CHO-S cells were transfected with the plasmid, selected for Blasticidin resistance, subcloned, and screened for production by ELISA for mIgG2a. The CD160-mIgG2a was purified from conditioned media by protein A sepharose chromatography.

### In vivo Treatment Protocol

Recipient mice were injected i.p. with CD160Ig or control Ig (murine IgG2a) on day 0 (0.5 mg) and on days 2, 4, 6, 8, and 10 (0.25 mg). CTLA4Ig (Abatacept, Bristol Myers Squibb) was given i.p on day 0 (0.5 mg) and on days 2, 4 and 6 (0.25 mg). Rapamycin was a generous gift of Joren Madsen (TBRC, Massachusetts General Hospital, Boston, Massachusetts, USA) and was administered i.p at 0.3 mg/kg for day 0–3.

### FACS Analysis

Splenocytes from WT and KO mice were obtained as single cell suspension and stimulated with various concentrations of ConA (0.1 and 1 mcg/ml) for 24. The percentage of CD160 expressing naive as well as effector/memory CD4^+^ and CD8^+^ T cells, characterized by a CD44^high^CD62L^low^ phenotype, was determined by flow cytometry. The percentages of effector/memory CD4^+^ and CD8^+^ cells in recipient’s spleen were measured at different time points. Flow cytometry was performed using a FACSCalibur flow cytometry system (Beckton Dickinson, San Jose, CA) and analyzed using CellQuest software (Beckton Dickinson, San Jose, CA). All antibodies were obtained from BD Pharmingen except of anti-CD160 PE (Clone BY55, Beckman Coulter).

### ELISPOT Assay

Splenocytes from recipient mice were obtained as single cell suspension and stimulated with irradiated (30 Gy) syngeneic, or allogeneic splenocytes (Balb/c). The technique for ELISPOT analysis has been described by our group and others [32]–[33] and was adapted to measure IFN-γ, and IL-5 secreting cells. All samples were tested in quadruplicate wells and frequencies are expressed as the number of spot forming cells (SFC) per 0.5×10^6^ splenocytes.

### Cytokine Analysis by LUMINEX Assay

The cell-free supernatants of the ELISPOT assay were harvested after 48 h and analyzed by multiplexed cytokine bead-based immunoassay using a 21-plex mouse cytokine detection kit (Upstate) according to the manufacturer’s instructions. All samples were tested in triplicate wells.

### CFSE Labeling

Splenocytes and LN cells from WT or CD28^−/−^ mice were obtained as single cell suspension and stained with CFSE (Molecular Probes, Portland, OR) as previously described [34]. Recipient BALB/c mice were sublethally irradiated (1000 rad) with a Gammacell Exactor (Kanata, Ontario, Canada). Each mouse received 6−8×10^7^ CFSE-labeled donor cells i.v. and was treated with CD160Ig or control Ig on day 0 and 2 (0.5 mg). Three days later, the *in vivo* proliferation of CFSE-labeled donor cells was evaluated by flow cytometric analysis and the responder frequency was calculated as previously reported [34]. The large number of cells transferred (6−8×10^7^ cells/mouse) and the time point examined (3 days) preclude homeostatic expansion of CFSE-labeled cells in the irradiated hosts, and cell division in this model is driven primarily by the host alloantigens [35].

### Statistics

Kaplan-Meier survival graphs were constructed and a log rank comparison of the groups was used to calculate *p* values. Student’s *t* test was used for comparison of means between experimental groups examined by FACS analysis or ELISPOT assay. Differences were considered to be significant at *p* values <0.05.

## Results

### Similar Expression Patterns of CD160 Receptor in WT and KO Mice

**Figure 1:**
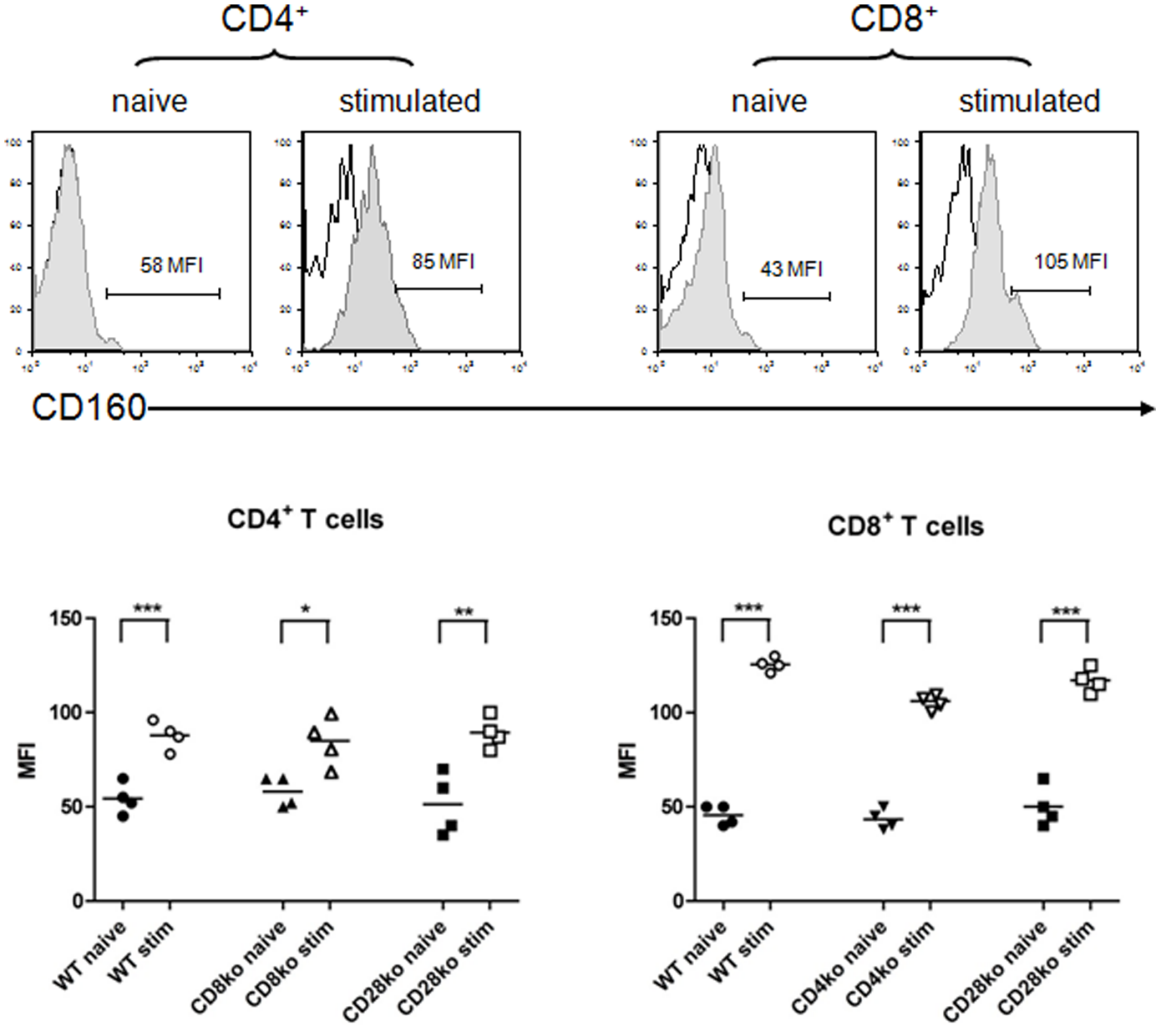
CD160 is expressed on CD8 and CD4 T cells. Naïve or stimulated (ConA) splenocytes from wildtype C57BL/6 (WT), CD4^−/−^, CD8^−/−^ and CD28^−/−^ mice were stained with anti-CD160. Mean fluorescence intensity of CD160 on naïve CD8^+^ and CD4^+^ T cells or CD8^+^ and CD4^+^ T cells expressing an effector/memory phenotype (CD44^high^CD62L^low^) was analyzed. **Upper Panel**: Representative dot plots of CD160 expressing cells (shaded histograms) in WT mice. **Lower Panel**: The histograms demonstrate the MFI of CD160^+^ cells of the overall CD4^+^ or CD8^+^ T cell population as the mean ± SEM of 3–5 independent experiments.

To characterize the expression of CD160, we stained splenocytes obtained form naïve WT and knockout (KO) mice with anti-CD160. Low level expression of CD160 was noted on both CD4^+^ and CD8^+^ T cells (2.8±1.3% and 2.3±1.8% respectively) as well as on NK cells (1.9±0.1%). To determine whether the expression of CD160 varies with the activation status of T cells we stimulated splenocytes with ConA *in vitro*. Subsequently, CD160 expression on CD4^+^ and CD8^+^ T cells, characterized by an effector/memory phenotype (CD44^high^CD62L^low^), was determined. We found that expression of CD160 was significantly upregulated on both CD4^+^CD44^high^CD62L^low^ (9.5±0.3%) and to a higher degree CD8^+^CD44^high^CD62L^low^ effector/memory T cells (14.5±1.5%), suggesting that CD160 expression is particularly induced after CD8^+^ T cell activation. Of notice, the expression pattern of CD160 did not differ between the WT mice and KO animals (Figure 1).

### Decreases Alloreactive T Cell Proliferation and IFN-γ Production

**Figure 2:**
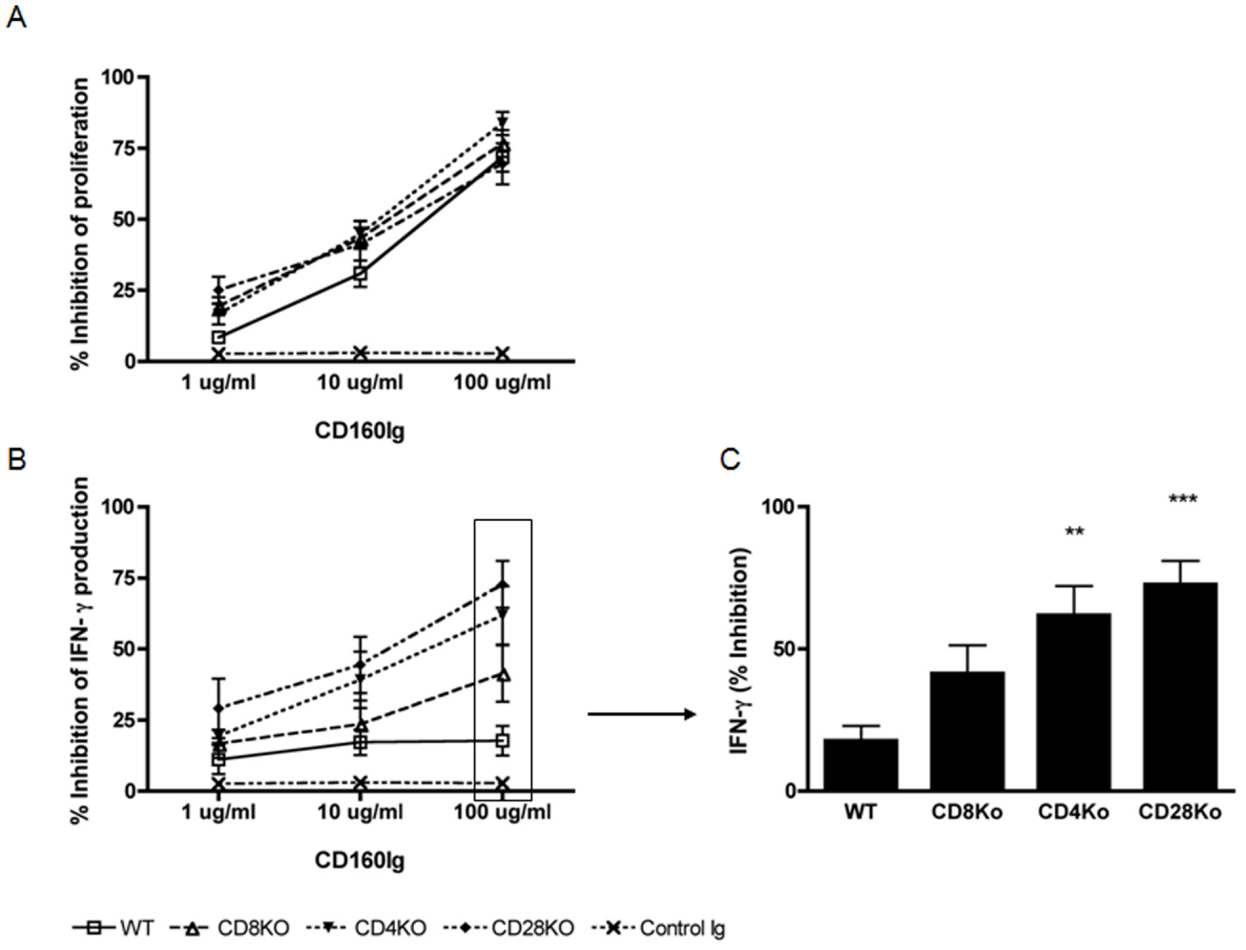
CD160Ig inhibits allospecific T cell proliferation and Th1 cytokine generation. Wildtype C57BL/6 (WT), CD4^−/−^, CD8^−/−^ and CD28^−/−^ mice (all H-2^b^) were sensitized with skins from fully missmatched BALB/c (H-2^d^) donors. Recipients splenocytes were harvested and cultured with irradiated donor splenocytes in the absence and in the presence of increasing concentrations of CD160Ig or control Ig. A) Cell proliferation is expressed as percent inhibition of CD160Ig treated cells compared with control Ig treated cells of that strain. Results are presented as the mean ± SEM of 3 experiments performed in triplicates. B) Frequency of alloreactive IFN-γ producing cells was quantified by ELISPOT assay after 24 hours. Percent inhibition of IFN-γ production of CD160Ig treated cells compared with control Ig treated cells of that strain is shown. Results shown are mean ± SEM values of 3 independent experiments performed in quadruplicate wells. **C.)** Statistics of percent inhibition of alloreactive IFN-γ production at 100 µg/ml CD160Ig/well.

To investigate the function of CD160, we generated a murine CD160Ig fusion protein, as described in the methods section. First we evaluated its role in alloreactive T cell proliferation *in vitro.* Since CD160 is upregulated after T cell activation we used splenocytes from sensitized WT and KO recipient mice, who had received a prior skin allograft from BALB/c mice. Splenocytes were stimulated with irradiated donor splenocytes (BALB/c) in the presence of increasing concentrations of CD160Ig or control Ig. Cell proliferation was measured by (^3^H)thymidine incorporation. As expected T cell proliferation was significantly reduced in CD4^−/−^ (24715±3990 cpm, n = 12; p = 0.0012), CD8^−/−^ (25964±3926 cpm, n = 12; p = 0.014), and CD28^−/−^ mice (16176±2676 cpm, n = 12; p<0.0001) as compared to WT mice (32633±3097 cpm, n = 12). CD160Ig strongly inhibited proliferation of both CD4^+^ and CD8^+^ T cells in a dose dependent manner. However, the inhibitory effect of CD160Ig was independent of CD28 costimulation, in that the proliferation of T cells from WT, CD4^−/−^ and CD8^−/−^ recipients was reduced to a similar degree as from CD28^−/−^ recipients (Figure 2A).

We then assessed the effect of CD160Ig on alloreactive IFN-γ production using an ELISPOT assay in the same culture conditions. Again IFN-γ-production was significantly reduced in CD4^−/−^ (190.9±13.7 spot forming cells (SFC), n = 12; p<0.0001), CD8^−/−^ (232.2±23.25 SFC, n = 12; p = 0.004), and CD28^−/−^ mice (198.2±35.42 SFC, n = 12; p = 0.0023) as compared to WT mice (414±39.25 SFC, n = 12). CD160Ig inhibited the alloreactive IFN-γ production moderately in a dose dependent manner in WT splenocytes (7, 15 and 17% respectively). Interestingly, its inhibitory effect was more pronounced in CD8^+^ than in CD4^+^ T cells, in that the maximal reduction in IFN-γ production was 60% in CD4^−/−^ (vs. WT; p<0.05) as compared to 46% in CD8^−/−^ recipients (vs. WT; ns). Notably, in the absence of CD28 costimulation we observed the maximal inhibition (>70%) of alloreactive IFN-γ production (vs. WT; p<0.0001) (Figure 2B and C).

### CD160Ig Prolongs Cardiac Allograft Survival in CD28^−/−^ and CD4^−/−^ Recipients

**Figure 3:**
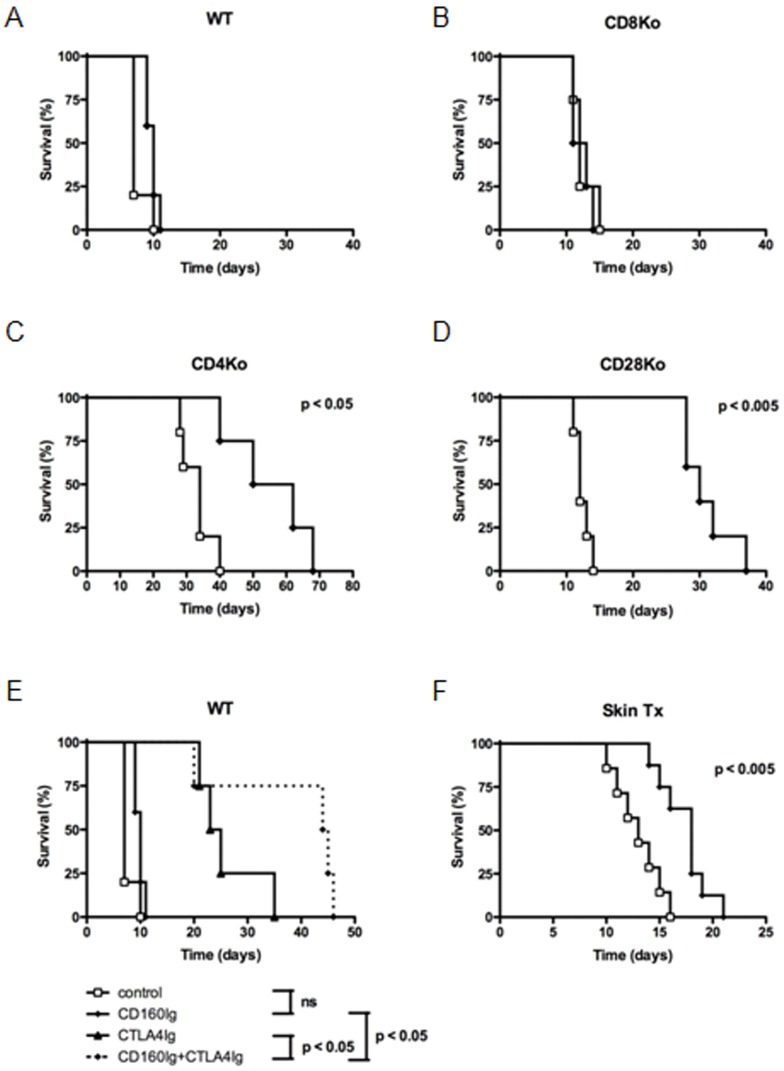
CD160Ig prolongs fully mismatched heart allograft survival in CD28^−/−^ and CD4^−/−^mice. A) C57BL/6 WT (n = 10); B) CD4^−/−^, (n = 10); C) CD8^−/−^ (n = 10); D) CD28^−/−^ (n = 10) or E) C57BL/6 WT treated with CTLA4-Ig (n = 5) received Balb/c heart grafts and were treated with CD160Ig (⧫; n = 5) or control Ig (⧫; n = 5); F) C57BL/6 WT (n = 5) received bm1 skin grafts and were treated with CD160Ig (⧫, n = 5) or control-Ig (⧫; n = 5); Survival is shown by Kaplan-Meier plots.

To delineate the role of the CD160:CD160L pathway *in vivo* we transplanted vascularized cardiac grafts from BALB/c donors into fully allogeneic WT and KO recipients which were treated with CD160Ig or control Ig. CD160Ig treatment had an insignificant effect on graft survival in WT (median survival time (MST) = 10 vs. 7 days, n = 5, ns, Figure 3A) and in CD8^−/−^ recipients (MST = 12 vs. 12 days, n = 5, ns, Figure 3B), while CD8^+^ T cell-mediated allograft rejection in CD4^−/−^ mice was significantly delayed (MST = 56 vs. 34 days, n = 5, p = 0.01, Figure 3C). In keeping with the *in vitro* data, CD160Ig treatment led to a striking prolongation in allograft survival in CD28^−/−^ mice (MST = 12 vs. 30 days, n = 5; p = 0.002) as shown in Figure 3D. To confirm these results in a more physiological context we went on and used CTLA4Ig in combination with CD160Ig in WT recipients. Indeed, combined treatment prolonged allograft survival to a greater extend as compared to CTLA4Ig treatment alone (MST = 44.5 vs. 24 days, Figure 3E).

To confirm the impact of CD160Ig on CD8^+^ T cell responses *in vivo* we studied its role in a MHC class I miss-matched skin allograft model (bm1 into B6), in which rejection is predominantly driven by CD8^+^ T cells. In accordance with its effect in CD4^−/−^ heart transplant recipients CD160Ig significantly prolonged skin allograft survival (MST = 18 vs. 13 days, n = 5; p<0.003), (Figure 3F).

### CD160Ig Inhibits IFN-γ and Promotes IL-5 Alloreactive Cytokine Production in CD28^−/−^ and CD4^−/−^ Recipients

**Figure 4:**
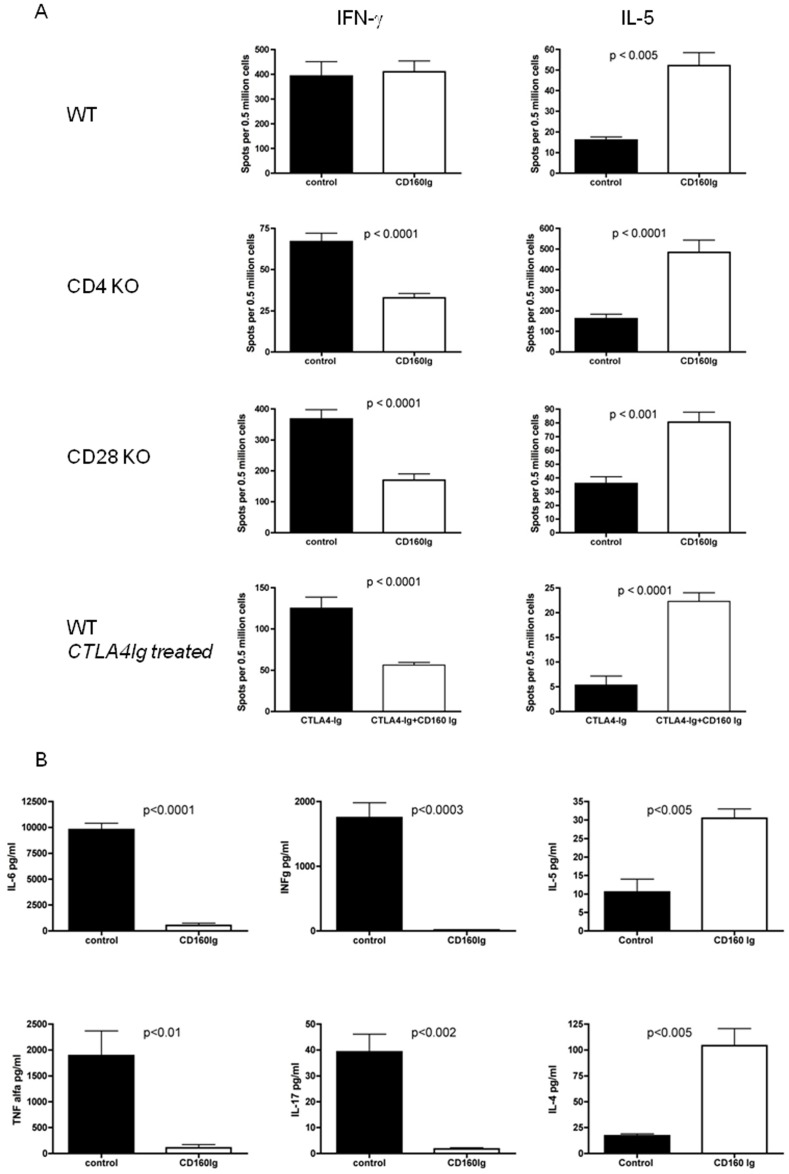
CD160Ig reduces allospecific Th1 cytokine generation in CD28^−/−^ and CD4^−/−^ mice. Heart grafts from BALB/c donors were transplanted into C57BL/6 WT, CD28^−/−^ or CD4^−/−^ recipients, which were treated with CD160Ig +/− CTLA4Ig or control Ig. Recipient splenocytes were isolated 10 days (WT recipients) or 14 days (CD28^−/−^ and CD4^−/−^ recipients, CTLA4Ig treated WT recipients) after transplantation, cultured with irradiated donor splenocytes. A) The frequency of alloreactive IFN-γ and IL-5 producing T cells was analyzed by ELISPOT. B). The concentration of TNF-α, IL-6, IFN-γ, IL-17, IL-4 and IL-5 was examined by Luminex Assay in the supernatants of the cultures with CD28^−/−^ responder cells. The data shown are pooled from 3–5 independent experiments performed in quadruplicate wells (Mean±SEM).

Given our *in vitro* and survival data we reasoned, that CD160Ig may prolong allograft survival in KO recipients by blocking the generation and/or function of alloreactive cytokine production *in vivo*. In order to explore this possibility, we measured the frequency of IFN-γ and IL-5 alloreactive T cells in WT, CD4^−/−^ and CD28^−/−^ recipients by ELISPOT assay. As shown in Figure 4A the frequency of alloreactive IFN-γ-producing T cells did not differ between CD160Ig treated and control Ig treated WT recipients (393±58 vs. 410±44 SFC, n = 12; ns). However, we noted a significant increase in the frequency of alloreactive IL-5 generating T cells (52±6.2 vs. 17±1.4 SFC; p>0.0005). Interestingly, the enhanced allograft survival in CD28^−/−^ recipients treated with CD160Ig or in WT recipients treated with a combination of CTLA4Ig and CD160Ig was associated with a robust reduction in the frequency of IFN-γ secreting alloreactive T cells (CD28^−/−^: 170±20 vs. 368±30 SFC, n = 30; p<0.0001; CTLA4Ig treated WT: 56±3 vs. 125±13 SFC, n = 20; p<0.0001), while the frequency of alloreactive IL-5 secreting T cells significantly increased (CD28^−/−^ recipients: 81±7 vs. 36±5 SFC; p<0.0001; CTLA4Ig treated WT: 5±2 vs. 22±2 SFC; p<0.0001). The same observation was true for CD4^−/−^ recipients after CD160Ig treatment (IFN-γ: 33±3 vs. 67±5 SFC; n = 20, p<0.0001, IL-5∶484±59 vs. 162±22; p<0.0001) indicating that prolongation of cardiac allograft survival with CD160Ig is associated with a switch in the cytokine profile of alloreactive T cells (Figure 4A).

Because crosslinking of CD160 has been shown to enhance the production of the cytokines IFN-γ, TNF-α and IL-6 [22]–[23] we studied the effect of CD160Ig on the production of these cytokines by Luminex Assay in CD28^−/−^ recipients. Confirming the results observed with the ELISPOT assay, CD160Ig treatment significantly inhibited IFN-γ, TNF-α, IL-6 and IL-17 production, while it increased the production of IL-4 and IL-5 (Figure 4B).

### CD160Ig Inhibits CD8^+^ Effector/Memory Cell Expansion in CD28^−/−^ Recipients

**Figure 5:**
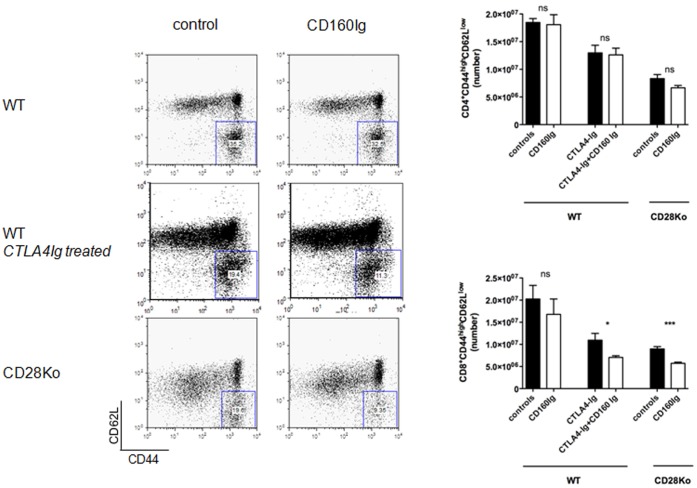
CD160Ig reduces effector/memory cell generation in CD28^−/−^ mice. Splenocytes from C57BL/6 WT, C57BL/6 WT treated with CTLA4Ig or CD28^−/−^ recipients of BALB/c hearts were harvested and CD4^+^ or CD8^+^ T cells were stained for the effector/memory phenotype, characterized as CD44^high^CD62L^low^. **Left Panel:** Representative dot plots of CD8^+^CD44^high^CD62L^low^ cells. **Right Panel:** The histograms demonstrate the frequency of CD44^high^CD62L^low^ cells as a percentage of the overall CD4^+^ or CD8^+^ T cell population as the mean ± SEM of 3–5 independent experiments.

To assess the effect of CD160Ig on generation effector/memory T cells we measured the percentage of CD4^+^ and CD8^+^ T cells manifesting a effector/memory phenotype (CD62L^low^CD44^high^) in WT, CD4^−/−^ and CD28^−/−^ recipients. In keeping with the ELISPOT data, no significant reduction in either CD4^+^ or CD8^+^ effector/memory T cells was observed in CD160Ig treated WT recipients. Interestingly, CD160Ig treatment did not alter the frequency of effector/memory CD8^+^ T cells in CD4^−/−^ recipients (5.1×10^6^±0.4×10^6^ cells vs. 5.4×10^6^±0.2×10^6^ cells n = 5, ns) despite the significant reduction in the frequency of alloreactive IFN-γ production. In contrast, the frequency of effector/memory CD8^+^ T cells was significantly reduced in CD160Ig treated CD28^−/−^ recipients (8.9×10^6^±1.5×10^6^ cells vs. 5.7×10^6^±0.8×10^6^%, n = 5, p<0.0001) as well as in CD160Ig plus CTLA4Ig treated WT recipients (11×10^6^±1.5×10^6^cells vs. 7×10^6^±0.3×10^6^cells, n = 4, p<0.05) (Figure 5), while the frequency of effector/memory CD4^+^ T cells was not affected. Furthermore, no effect of CD160Ig on NK cells could be observed in any group as assessed by quantification of the total NK (NK1.1^+^CD3^−^CD122^+^) cell numbers in various lymphoid organs of recipient mice and the expression of various activation (CD25 and CD69) or adhesion molecules (CD44) on NK cells by FACS analysis.

### CD160Ig does not Alter Expression of CD160 or MHC Class I on APCs

Recently HVEM was identified as the signaling ligand for CD160 [24]. However, CD160 has been shown to bind both classical and non-classical MHC class-I molecules with low affinity [16], [19], [22]–[23]. Therefore, we examined the level of expression of MHC class I molecule on the surface of CD19^+^ B cells, CD11c^+^ dentritic cells and CD11b^+^ monocytes derived from naïve, as well as CD160Ig treated and control Ig treated WT and KO mice at baseline and time of rejection. As expected, flow cytometry analysis revealed that MHC class I was expressed by naïve B cells, dentritic cells and monocytes and the level of expression significantly increased during the alloresponse as compared to baseline on all three cell types (data not shown). Importantly, MHC class I molecule expression did not differ between WT and the various KO mice at any time point excluding, that a limited amount of antigen and/or low level of TCR/pMHC interaction during the alloresponse was the reason for the observed differences in allograft survival between the groups. Furthermore, we were unable to observe marked differences in the MHC class I expression between CD160Ig and control Ig treated recipients (Figure S1).

### CD160Ig does not Alter Cytolytic Activity of CD8^+^ T Cells and NK Cells in CD28^−/−^ Recipients

Previous studies have shown, that CD160 enhances the cytolytic activity of CD8^+^ T cells and NK cells [16], [22]–[23]. Therefore, we examined the effect of CD160Ig on cytotoxic cell subsets *in vivo* by measuring the frequency of GranzymeB producing alloreactive cells by ELISPOT in WT, CD4^−/−^ and CD28^−/−^ recipients. However, CD160Ig treatment had an insignificant effect on alloreactive GranzymeB production in all groups suggesting, that CD160Ig did not influence cytolytic activity of CD8^+^ T cells and NK cells (data not shown).

### CD160Ig Reduces Alloreactive CD8^+^ T Cell Proliferation in vivo in the Absence of CD28

**Figure 6:**
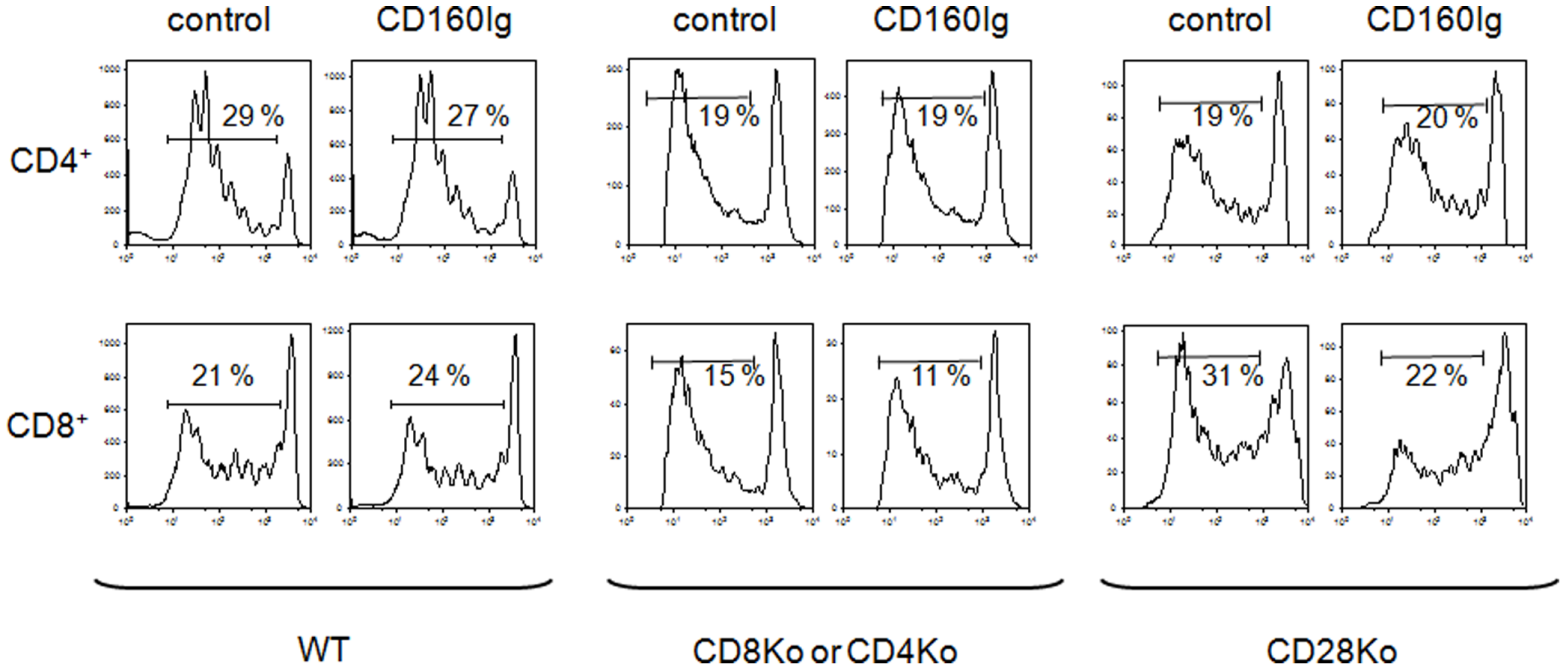
CD160Ig decreases in vivo proliferation of CD28 deficient CD8^+^ T cells. CFSE-labeled splenocytes from C57BL/6 WT, CD8^−/−^, CD4^−/−^ or CD28^−/−^ mice (6−8×10^7^) were injected into irradiated BALB/c hosts and treated with CD160Ig or control Ig. The precursor division frequency of CD4^+^ and CD8^+^ T cell subsets in the host spleen was analyzed 3 days later by FACS and calculated as described in Materials and Methods. Representative histograms from one of 3 experiments are shown.

The expansion of alloreactive T cell clone size is a key event in initiation of the rejection process. To examine the impact of CD160Ig on T cell proliferation *in vivo* CFSE labeled leucocytes from either WT or CD28^−/−^ mice were adoptively transferred into irradiated allogeneic BALB/c hosts and CD4^+^ and CD8^+^ T cell proliferation was assessed by FACS analysis 3 days thereafter. In the presence of CD28 costimulation, CD160Ig did not inhibit the in vivo proliferation of CD4^+^ (30.1±4.4 vs. 30±4% precursor division frequency (PDF), n = 5) and CD8^+^ T cells (28±1.4 vs. 27.4±2.2% PDF, n = 4). However, CD160Ig significantly reduced the *in vivo* proliferation of CD28 deficient CD8^+^ T cells (26±3.8 vs. 19±2.7% PDF, n = 5, p = 0.01) and, although to a lesser degree, that of CD28 deficient CD4^+^ T cells (17±2.2 vs. 14.7±1.7% PDF, n = 5, p = 0.04) (Figure 6).

### Clinical Relevance

Rapamycin has been reported to synergize with a variety of strategies targeting T cell costimulatory pathways [31], [36]. To study the clinical relevance of CD160Ig we evaluated its effect in the combination with a subtherapeutic dose of rapamycin in WT recipients. While CD160Ig alone had no effect on allograft survival in WT recipients, subtherapeutic doses of rapamycin resulted in a moderate prolongation of allograft survival in WT mice (MST 29 vs. 7 days, n = 5; p<0.002). However, combination of both resulted in synergistic prolongation of allograft survival (MST 46 days, n = 5; p<0.001) not seen with either agent alone. The prolonged allograft survival after rapamycin treatment was associated with a significant reduction in the alloreactive Th1 cytokine production, as assessed by IFN-γ ELISPOT, and generation of effector/memory CD8^+^ T cells. However, the addition of CD160Ig did not further reduce the alloreactive Th1 effector response, but significantly enhanced the number of alloreactive IL-4 and IL-5 producing cells, indicating again, that CD160Ig is associated with a marked Th2 switch in the cytokine profile of alloreactive T cells (Figure S2).

## Discussion

This is the first study establishing the *in vivo* function of the novel T cell costimulatory pathway CD160:CD160L in alloimmune responses. Our data clearly demonstrate that the CD160:CD160L pathway plays an important role in CD28-independent CD8^+^ T cell alloimmune responses. CD160 is expressed on a small percentage of CD4^+^ and CD8^+^ T cells and is predominantly upregulated after CD8^+^ T cell activation. CD160Ig inhibits the alloreactive T cell proliferation and IFN-γ production *in vitro* with the most profound effect on CD8^+^ T cells and lymphocytes lacking CD28 expression. CD160Ig consequently prolongs allograft survival in CD4^−/−^, CD28^−/−^ as well as CTLA4Ig treated WT recipients. The prolongation in allograft survival is associated with reduced alloreactive CD8^+^ T cell proliferation, diminished generation of effector/memory CD8^+^ T cells and switch in the cytokine profile of alloreactive T cells. Interestingly, CD4^+^ T cell functions appear to be relatively insensitive to the effects of CD160Ig *in vivo* in our model.

It is well recognized that blocking the CD28:B7 costimulatory pathway is effective in inducing tolerance in some rodent models [2]–[6], but is not sufficient to block transplant rejection in more stringent models [7]. Effector/memory responses [14] and/or CD8^+^ T cell responses [10]–[13] appear to be less dependent on CD28 costimulation and represent a significant barrier to the development of tolerance to alloantigen [14], [37]. Our results establish an important role for CD160:CD160L pathway in CD8^+^ effector/memory T cell responses *in vivo*, especially in the absence of CD28 costimulation, as seen by the suppressed alloreactive IFN-γ cytokine generation and contraction in the size of the effector/memory cell population (CD44^hi^CD62L^low^) of CD8^+^ T cells. These data are in keeping with previously published findings that expression of murine CD160 is largely restricted to cytotoxic NK cells and a subset of cytotoxic CD8^bright+^ T cells [17]–[18], [38] as well as CD8^+^ effector/memory T cells [19]–[20]. Furthermore, CD160 has been shown to act as a co-receptor in TCR signal transduction in CD8^+^ T cells [28] leading to the expansion of CD28^−^ T lymphocytes [16], [28], [38].

Recent data indicate that the membrane-bound CD160 molecule can be cleaved from the cell surface upon activation and released as a soluble form (sCD160) which serves to limit CD8^+^ effector functions by binding to MHC class-I on target cells *in situ*. This provides a physiological mechanism to control CD8^+^ CTL activity [20], [26]. sCD160 mediated down-modulation of cytolytic activity is not restricted to cytotoxic T cell clones, but is also effective on allogeneic stimulated T cells [26]. However, in our study CD160Ig inhibited alloreactive CD8^+^ T lymphocyte proliferation and IFN-γ production but did not alter the cytolytic activity. However, CD160 mRNA expression has been demonstrated on exhausted CD8 T cells in the late stage of chronic viral infection, which coincides with loss of CTL function [39]–[41]. Thus the effect of CD160Ig seen in our model might be the result of blocking a co-stimulatory signal in late stage differentiated CD8+ T cells, leading to a diminished proliferation, expansion and differentiation rather than affecting their cytotoxic capacity.

The mechanism by which CD160Ig prolongs allograft survival could also be due to both reduction in pro-inflammatory T cells (alloreactive IFN-γ cells) and induction of anti-inflammatory alloreactive IL-5 cells, which would be in agreement with several studies showing that tolerance induction by costimulatory blockade is sometimes associated with a state of “immune deviation” toward Th2 cell function [5]. However, whether a Th2 switch per se can mediate transplant tolerance remains controversial [42]. The effect of Th2 deviation in promoting graft survival seems to depend on the alloreactive T cell clone size [43]–[44], as shown after CD160Ig treatment.

However, it is increasingly recognized, that active regulation to maintain self-tolerance and prevent alloimmune responses is not only due to CD4^+^ regulatory T cells (Tregs), but is also dependent on the presence of CD8^+^ Tregs [45]. Among others CD8^+^CD28^−^ Tregs have been described both in humans and CD28^−/−^ mice [46]–[48]. Recent evidence indicates a potentially important relationship between immune modulatory Th2 cytokines and Tregs. The more pronounced effect of CD160Ig in the absence of CD28 costimulation might therefore be due to an enhanced active regulation.

Despite the inherent complexity of the costimulatory pathway interactions, selective blockade of individual molecules remains an attractive target as part of a multifaceted approach, as the phase II clinical study using CTLA4Ig in renal transplant recipients successfully showed [49]. Combination therapy of costimulatory blockade with CTLA4-Ig, MR1 [50] or rapamycin [51] has been shown to prolong graft survival. In the current study, we demonstrate the synergistic effect of CD160Ig when combined with rapamycin and CTLA4-Ig in prolonging allograft survival. We anticipate that targeting the CD160:CD160L pathway in combination with theses reagents may provide clinical benefits through their role as part of a less toxic maintenance strategy.

In conclusion, CD160 signaling is particularly critical for CD28-independent effector/memory CD8^+^ alloreactive T cell activation *in vivo* and therefore the novel fusion protein, CD160Ig, may prove to be a promising reagent in strategies for prevention of allograft rejection in conjunction with B7/CD154 T cell costimulatory blockade.

## Supporting Information

Figure S1 CD160Ig has no effect on MHC class I expression. A) CD11c^+^ dentritic cells from naïve WT, CD4^−/−^, CD8^−/−^ and CD28^−/−^ mice were stained for the expression of MHC class I molecules. B) CD11c^+^ dentritic cells from CD28^−/−^ recipients of BALB/c hearts were stained for the expression of MHC class I molecules at baseline (day 0) and on day 14 and 30 (time point of rejection) in treated and untreated recipients. The histograms demonstrate the frequency MHC class I positive cells as a percentage of the overall CD11c^+^ T cell population as the mean ± SEM of 3–5 independent experiments.
(TIF)

Figure S2 CD160Ig in combination with rapamycin prolongs fully mismatched heart allograft survival WT mice. C57BL/6 WT (n = 5) mice received Balb/c heart grafts and were treated with CD160Ig +/− subtherapeutic dosis of rapamycin (0.3 mg/kg for days 0–3) as described in Materials and Methods. A) Kaplan-Meier plots demonstrate allograft survival B) The histogram demonstrates the frequency of CD44^high^CD62L^low^ cells as a percentage of the overall CD8^+^ T cell population isolated on day 14 after transplantation as the mean ± SEM of 3–5 independent experiments. C - E) The histograms demonstrate the alloreactive IFN-γ (C), IL-4 (D) and IL-5 (E) production, as assessed by ELISPOT on day 14 as the mean ± SEM of 3–5 independent experiments.
(TIF)
