# Supplementary figures and images for: CD160Ig Fusion Protein Targets a Novel Costimulatory Pathway and Prolongs Allograft Survival
Source: PLoS One. 2013 Apr 4;8(4):e60391. doi: 10.1371/journal.pone.0060391 (PMC3617215; doi:10.1371/journal.pone.0060391)

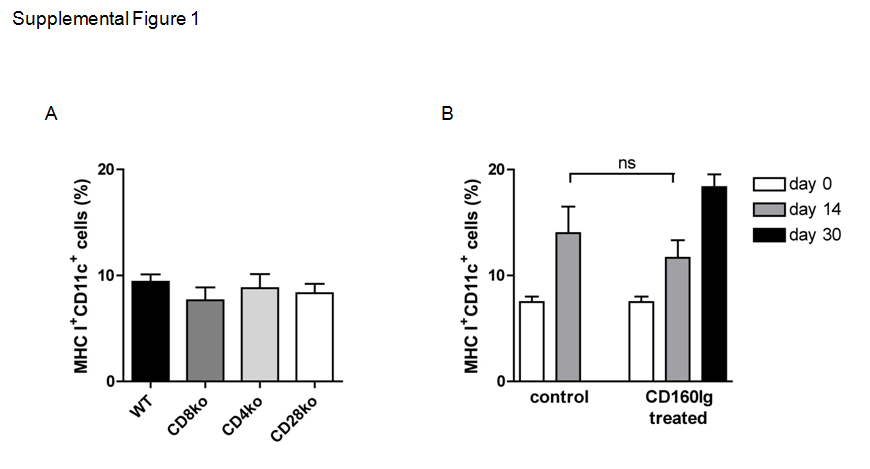

Supplement: Figure S1 — CD160Ig has no effect on MHC class I expression. A) CD11c+ dentritic cells from naïve WT, CD4−/−, CD8−/− and CD28−/− mice were stained for the expression of MHC class I molecules. B) CD11c+ dentritic cells from CD28−/− recipients of BALB/c hearts were stained for the expression of MHC class I molecules at baseline (day 0) and on day 14 and 30 (time point of rejection) in treated and untreated recipients. The histograms demonstrate the frequency MHC class I positive cells as a percentage of the overall CD11c+ T cell population as the mean ± SEM of 3–5 independent experiments. (TIF) [file pone.0060391.s001.tif]

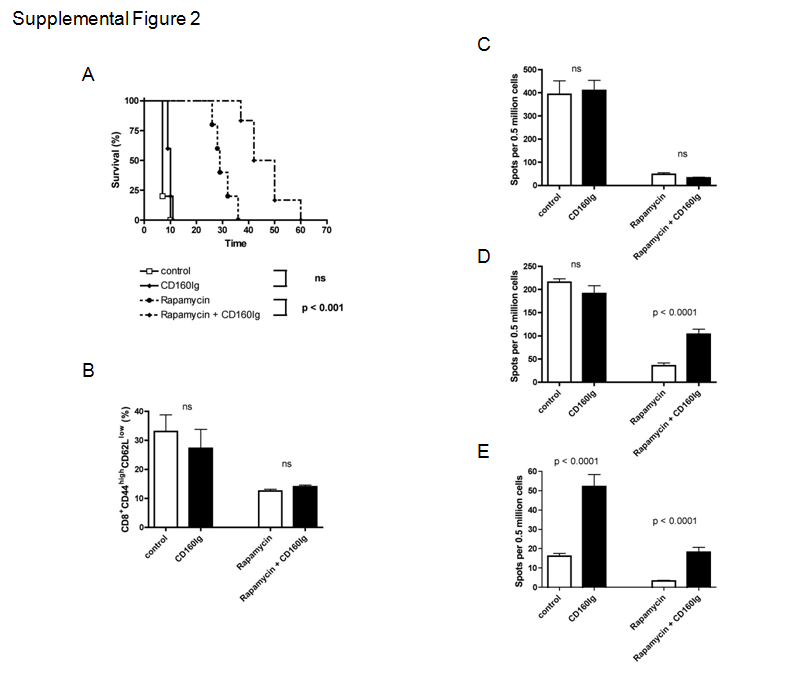

Supplement: Figure S2 — CD160Ig in combination with rapamycin prolongs fully mismatched heart allograft survival WT mice. C57BL/6 WT (n = 5) mice received Balb/c heart grafts and were treated with CD160Ig +/− subtherapeutic dosis of rapamycin (0.3 mg/kg for days 0–3) as described in Materials and Methods. A) Kaplan-Meier plots demonstrate allograft survival B) The histogram demonstrates the frequency of CD44highCD62Llow cells as a percentage of the overall CD8+ T cell population isolated on day 14 after transplantation as the mean ± SEM of 3–5 independent experiments. C - E) The histograms demonstrate the alloreactive IFN-γ (C), IL-4 (D) and IL-5 (E) production, as assessed by ELISPOT on day 14 as the mean ± SEM of 3–5 independent experiments. (TIF) [file pone.0060391.s002.tif]
